# Dictionary-based matching graph network for biomedical named entity recognition

**DOI:** 10.1038/s41598-023-48564-w

**Published:** 2023-12-08

**Authors:** Yinxia Lou, Xun Zhu, Kai Tan

**Affiliations:** 1 School of Artificial Intelligence, Jianghan University, Wuhan, 430056 China; 2 State Key Laboratory of Estuarine and Coastal Research, East China Normal University, Shanghai, 200241 China

**Keywords:** Computational models, Data mining, Machine learning

## Abstract

Biomedical named entity recognition (BioNER) is an essential task in biomedical information analysis. Recently, deep neural approaches have become widely utilized for BioNER. Biomedical dictionaries, implemented through a masked manner, are frequently employed in these methods to enhance entity recognition. However, their performance remains limited. In this work, we propose a dictionary-based matching graph network for BioNER. This approach utilizes the matching graph method to project all possible dictionary-based entity combinations in the text onto a directional graph. The network is implemented coherently with a bi-directional graph convolutional network (BiGCN) that incorporates the matching graph information. Our proposed approach fully leverages the dictionary-based matching graph instead of a simple masked manner. We have conducted numerous experiments on five typical Bio-NER datasets. The proposed model shows significant improvements in F1 score compared to the state-of-the-art (SOTA) models: 2.8% on BC2GM, 1.3% on BC4CHEMD, 1.1% on BC5CDR, 1.6% on NCBI-disease, and 0.5% on JNLPBA. The results show that our model, which is superior to other models, can effectively recognize natural biomedical named entities.

## Introduction

Biomedical named entity recognition (BioNER) is a critical task in biomedical text mining that aims to identify various existing biomedical entities such as genes, proteins, chemicals, and diseases from text. BioNER is useful for extracting new genes and other important biomedical entities from research articles^1^. Additionally, BioNER serves as a foundational step for other essential tasks like relation extraction^2^ and knowledge base completion^3^. The accuracy of BioNER tools remains a crucial factor in the performance of biomedical text mining pipelines^4^. Improving the accuracy of BioNER is crucial for advancing biomedical research and developing new treatments and therapies for a wide range of diseases.

BioNER is often regarded as a sequence labeling problem. Owing to the rapid development of deep learning, many neural structures^5,6^ have been proposed to address this task. Basic neural networks such as long short-term memory network (LSTM) and its variant bidirectional LSTM (BiLSTM) achieve better performance compared with traditional feature-based approaches^7^. Other neural structure types like convolutional neural network (CNN)^8^ and Transformers^9^ can also be selected as alternatives and obtain comparable results. Taking the entity head-tail boundary detection as an auxiliary task enhances named entity recognition^10^. Inspired by the excellent ability of Bidirectional Encoder Representation from Transformers (BERT) on text representation, researchers proposed biomedical BERT (BioBERT) which is pre-trained on biomedical corpus^11^. Despite the great success achieved through deep learning methods, there remain some unresolved issues. One prominent shortcoming is that these models rarely integrate human knowledge. The deep neural networks often attempt to directly learn features from large scale labeled data. However, there also exists a substantial number of entities that rarely or even do not occur in the training set. Thus, the data-driven deep learning methods usually cannot handle such cases well.

**Figure 1:**
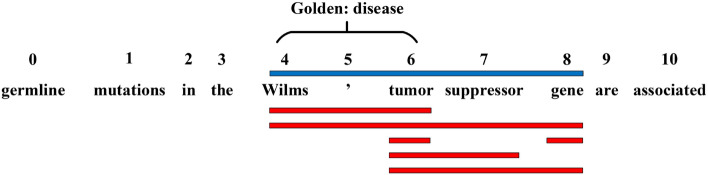
A typical sample of biomedical named entity recognition task. Blue bar indicates the mask sequence generated by simple masked manner. Red bars represent all the possible entities appearing in the dictionary. Golden entity is ‘Wilms ’ tumor’ with type ‘disease’.

To address the above challenge, one approach is to leverage extra dictionary information. Biomedical dictionaries are already widely employed in many neural models^12,13^ as a supplementary information gathered from a simple masked manner, which normally will be fed into the input. Specifically, to fully leverage external dictionary resources, many works introduce position features of words within the lexicon, including the word’s beginning (B), middle (M), or end (E). Wang et al. ^12^ introduced a position-dependent entity type feature by attaching position labels (BME) to the rear of each word in the lexicon.^13^ presented a relational graph to utilize the position information of word tokens by adding the boundary information of words to the edges that link lexicon words and tokens. However, the works mentioned above that incorporate dictionary information and its positions have shown limited improvement in performance. Figure 1 illustrates that a single text can contain multiple interacting entities from biomedical dictionaries, with three types of relationships among them: overlapping, nested, and disjoint. Traditional models using the masked approach can only handle the disjoint situation. As depicted in Figure 1, the golden entity is only a sub-sequence of the masked entity but exists in the dictionary (one of the red bars). It also has complex spatial relationships with other red bars. For overlapping and nested situations, we need to devise a coherent structure to maintain all matching information (red bars) and handle the redundant parts among these entities strategically.

**Figure 2:**
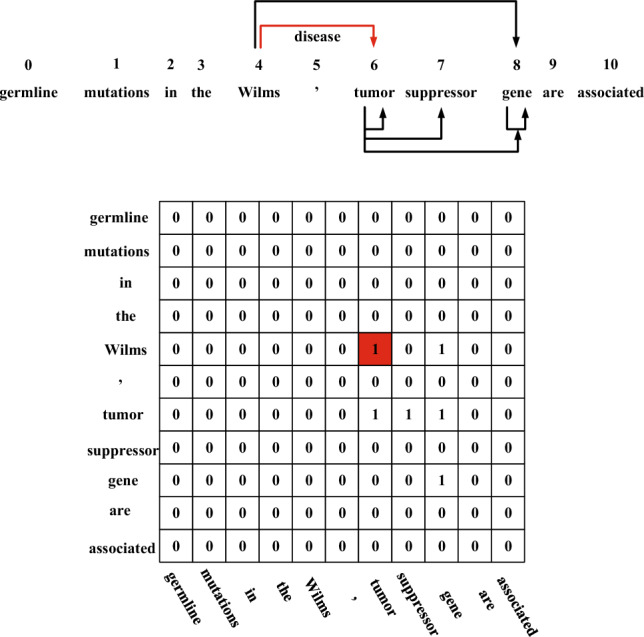
A sample demonstrates how entities appearing in the dictionary are transformed into unique connections. The matrix is an adjacent matrix. Red arrow or block means the golden entity.

In this study, we propose a dictionary-based matching graph network (DMGN) to process all entities appearing in the biomedical dictionary accurately. As shown in Fig. 2, each entity can be uniquely defined by a tuple including a start and end point, which can be treated as a connection from the start point to the end point. We can then construct a directional graph using these connections. To describe the graph, we introduce the graph convolutional network (GCN)^14^ to our method. In particular, we use the bidirectional GCN (BiGCN) to encode both the forward and backward graphs. This method computes the start and end information of each word when forming an entity. We also use BiLSTM and BioBERT as our basic encoders to represent the text information. The results on five datasets demonstrate that DMGN significantly improves the performance compared to methods using a masked manner.

## Background

### Long short-term memory (LSTM)

LSTM takes a vector sequence $[x_1,x_2,...]$ as the input and outputs hidden states $[h_1,h_2,...]$. LSTM consists of three main gates, including input gate, output gate and forget gate that precisely control the message flow through each inner module. In general, we use the sigmoid function as the activation function, which restricts the output value between zero and one.. The main procedure is formulated as follows:

1 $$\begin{aligned} i_t&=sigmoid(W_i[h_{t-1},x_t]+b_i) \end{aligned}$$

2 $$\begin{aligned} f_t&=sigmoid(W_f[h_{t-1},x_t]+b_f) \end{aligned}$$

3 $$\begin{aligned} o_t&=sigmoid(W_o[h_{t-1},x_t]+b_o) \end{aligned}$$

4 $$\begin{aligned} {\tilde{h}}_t&=Tanh(W_h[h_{t-1},x_t]+b_h) \end{aligned}$$

5 $$\begin{aligned} c_t&=f_t*c_{t-1}+i_t*{\tilde{h}}_t \end{aligned}$$

6 $$\begin{aligned} h_t&=o_t*Tanh(c_t) \end{aligned}$$

$c_t$ is the memory state and $h_0$ is initialized to zeros, where *t* represents the time step. The parameters $W_i,W_f,W_o,W_h$ and $b_i,b_f,b_o,b_h$ are all trainable. The input gate $i_t$ controls the weight of the last hidden vector to form the mid vector ${\tilde{h}}_t$. The forget gate $f_t$ controls the proportion between the mid vector ${\tilde{h}}_t$ and the last hidden vector $h_{t-1}$ to obtain the current hidden vector $h_t$. The output gate $o_t$ controls the weight of the current memory $c_t$.

The LSTM architecture described above can only process the input in one direction. The bi-directional long short-term memory (BiLSTM) model improves the LSTM by feeding the input to the LSTM network twice, once in the original direction and once in the reverse direction. Outputs from both directions are concatenated to represent the final output. This design allows the model to detect dependencies from both previous and subsequent words in a sequence.

### Graph convolutional network (GCN)

GCN^14^ is a specialized neural network designed for processing graph structured data. We can denote the nodes as $H=\{h_1,h_2,...\}$ in the graph, and $H \in R^{N\times E}$. *N* is the number of the nodes, and *E* is the size of the hidden vector $h_i$, where $i\in [1,N]$. The graph embeddings of the nodes can be updated as follows:

7 $$\begin{aligned} H_{t+1}=Tanh(\frac{1}{|D|}DH_{t}W) \end{aligned}$$

$D \in R^{N\times N}$ is the adjacent matrix of the graph. |*D*| is a normalization function related to the adjacent node number. $W \in R^{E\times E}$ is a trainable weight. *t* denotes the current time step. $H_t \in R^{N\times E}$ is a collection of node embeddings at the *t*-th step, where $H_0$ is initialized as *H*. It is worth noting that node embeddings are iteratively updated by their neighboring nodes, which expands the influence range in each independent step.

### BioBERT

BioBERT^11^ shares the same structure with BERT, a novel contextual representation method based on a pre-training procedure on Transformers^9^. BERT uses a masked language model that predicts randomly masked words in a sequence, making it suitable for learning bidirectional representations. BERT has shown prominent performance on many natural language processing (NLP) tasks.^15^ showed that this augmentation is also suitable for biomedical text mining, owing to the similarly complex relationships among biomedical terms.

## Approach

**Figure 3:**
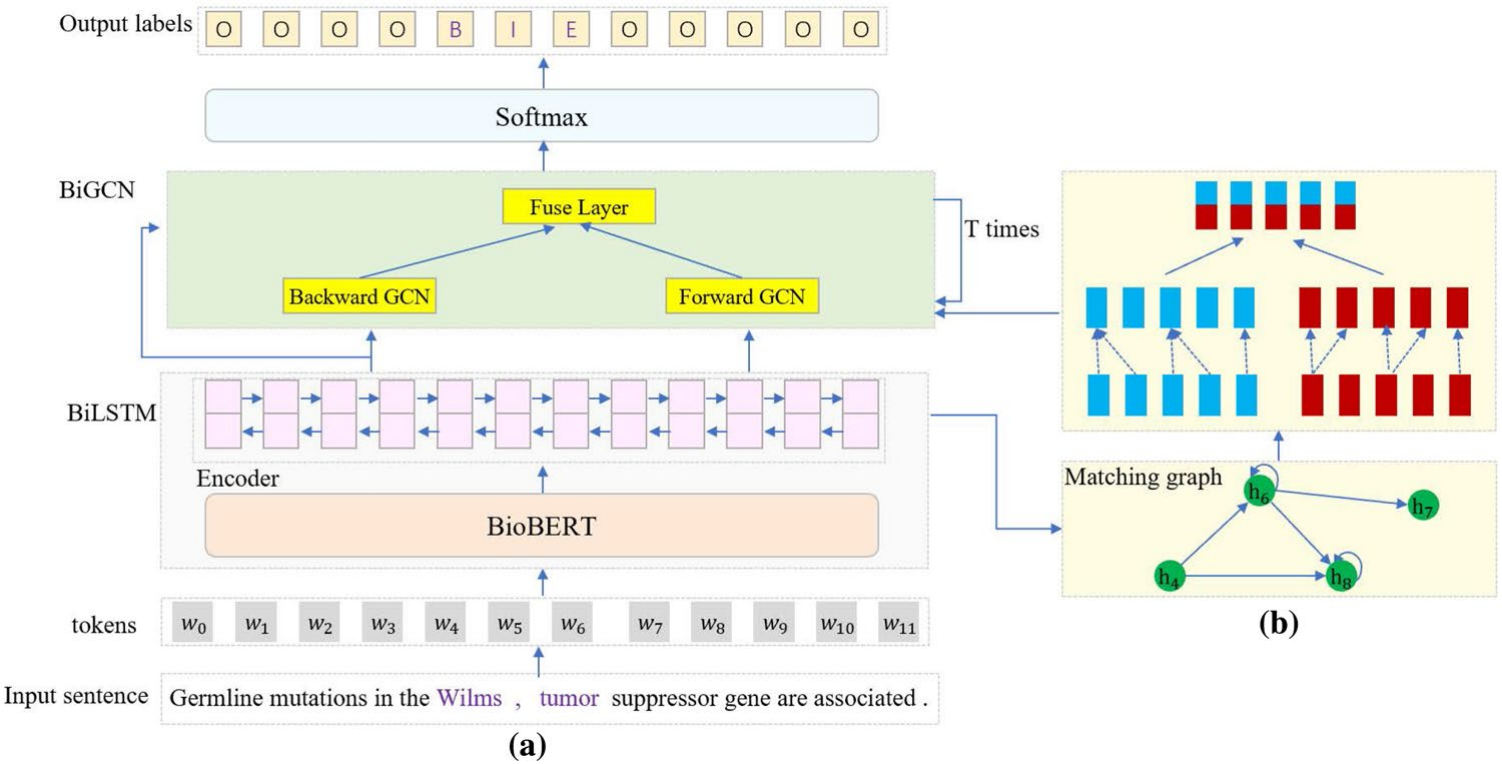
(**a**) A brief demonstration of our model. BiLSTM and BioBERT are utilized as basic encoders, dictionary-based matching graph and its reverse version are encoded by BiGCN. This module can be repeated for multiple times. (**b**) A more detailed demonstration of matching graph and BiGCN, two GCNs (blue and red ones) have completely reverse graphs.

The elaborate architecture of our model is exhibited in Fig. 3. We feed the adjacent matrix in Fig. 2 and its reverse version into the BiGCN module. It encodes the dictionary-based matching graph information in both forward and backward directions. *T* is a hyper-parameter indicating the number of layers in BiGCN and is determined according to the experiments. A residual connection is introduced to BiGCN to maintain the original hidden outputs of BiLSTM.

### Problem definition

Given an input text sequence $X=\{w_0,w_1,...\}$, the system is required to output the corresponding label sequence $Y=\{y_0,y_1,...\}$. Each word is annotated with a specific tag in the BIOES tag-set. For example, the output of ‘Wilms ’ tumor suppressor gene’ should be ‘B-disease I-disease E-disease O O’, where ‘O’ means a non-entity token and ‘disease’ indicates a disease type.

### BioBERT and BiLSTM encoder

Biomedical Bidirectional Encoder Representations from Transformers (BioBERT) have already shown great ability in providing contextual representations for multiple tasks in different domains. We use the PieceTokenizer to further tokenize words into subwords. These subwords are later combined to reconstruct the original words by applying a sum operation over their corresponding subword representations.

We use $w_{j}$ to represent the *j*-th word. Assume that all tokens are already processed by BioBERT, then $b_{j}$ denotes the *j*-th word BioBERT embedding. Bidirectional LSTMs^16,17^ are applied for the next encoder. *L* is the number of input words. Then, we can get the output states by following procedure:

8 $$ {h\,}_{i}^{f}=LSTM_{forward}([b_i,{h\,}^{f}_{i-1}]) $$

9 $${h\,}_{i}^{b}=LSTM_{backward}([b_i,{h\,}^{b}_{i+1}]) $$

Let $i \in [1, L]$ be the index of the word, where *L* is the length of the input sequence. We use $LSTM_{forward}$ and $LSTM_{backward}$ to represent two LSTMs with opposite directions, which process the input sequence in the forward and backward directions, respectively. At each position *i*, the concatenation of the *i*-th forward and backward hidden states, denoted as $h_i = [h_i^{f}, h_i^{b}]$, is used as the *i*-th output state. The collection of all output states is denoted by $H=\{h_1,...,h_L\}$.

### Bidirectional Graph Convolutional Network (BiGCN)

As shown in Fig. 2, the spans of entities appearing in the dictionary can be transformed into connections of a directional graph. We can thus obtain the adjacent matrix and feed it to BiGCN to encode the graph information. We use both forward and backward directions to encode the start and end information of each word when forming an entity.

**BiGCN** Dictionary-based matching graph in Fig. 2 defines the connection paths among the words. We design a bidirectional GCN to encode the graph information in both directions instead of a single GCN that ignores the connection direction . The whole computation are formulated as two following GCNs:

10 $$\begin{aligned}{} & {} Q^t_{out}=Relu\left(\frac{1}{|A^{out}|}A^{out}H_tW_{out}\right) \end{aligned}$$

11 $$\begin{aligned}{} & {} Q^t_{in}=Relu\left(\frac{1}{|A^{in}|}A^{in}H_tW_{in}\right) \end{aligned}$$

$A^{out}$ is the main adjacent matrix in Fig. 2, and $A^{in}$ is the reverse version of $A^{out}$. $H_i \in R^{L\times h}$ is initialized by $H_0=H$, which are the outputs of BiLSTM. *t* is the current time step. $Q^t_{out}$ and $Q^t_{in}$ are forward and backward intermediate node embeddings of the *t*-th step, respectively. |...| means normalize function.

12 $$\begin{aligned}{} & {} H_{t+1}=Norm(H_t+Relu([Q^t_{out},Q^t_{in}]W_O)) \end{aligned}$$

13 $$\begin{aligned}{} & {} H_{t+1}=BiGCN(H_t) \end{aligned}$$

*BiGCN* denotes the overall procedure of Equations (4)–(6). We merge the representation of two directions in each iteration, while other similar methods conduct the merging only in the last iteration. $W_{in},W_{out},W_O$ are all trainable coefficients. We also introduce residual connection to Eq. (6), considering the original encoding information of *H*.

### Loss function

We can get $H_T$ from Eq. (7) after *T* iterations, where *T* also indicates the layer size of BiGCN. $H_T$ can be decomposed as $\{h^T_1,...,h^T_L\}$, where $h^T_i$ represents the *i*-th word graph embedding of *T*-th time step. The loss function is formulated as follows:

14 $$\begin{aligned}{} & {} p(y_i|X)=softmax(h^T_iW_p) \end{aligned}$$

15 $$\begin{aligned}{} & {} loss=\sum _{i=1}^{L}-log(p(y_i=y^l_i|X)) \end{aligned}$$

*X* is the input text and $W_p$ is a trainable parameter. $y_i$ indicates the label of the *i*-th token. $p(y_i|X)\in R^C$ indicates the label probability distribution of the *i*-th token, where *C* is the label number. $y^l_i$ denotes the golden label of the *i*-th token. Our main goal is to minimize *loss* function using the stochastic gradient descent (SGD) algorithm.

**Table 1:** Biomedical NER datasets used in our experiments.

Dataset	Train	Dev	Test	Entity types
BC2GM	12574	2519	5038	Gene/Protein
BC4CHEMD	30682	30639	26364	Chemical
BC5CDR	4560	4581	4797	Chemical, disease
NCBI-Disease	5424	92	940	Disease
JNLPBA	18534	1932	4243	Gene/protein, cell

## Experiments

### Datasets

We conduct our experiments on five mainstream biomedical datasets from^18^. The overall detailed statistics are listed in Table 1. BIOES tag-set^19^ is introduced to annotate golden entities for these datasets. For example, *B-Disease* indicates a beginning token of a disease entity. *I-Disease* indicates an inner token of a disease entity. *O* indicates a non-entity token. *E-Disease* indicates the end token of a disease entity. *S-Disease* indicates a single token of an entire disease entity. We briefly describe those five datasets as follows:

- *BC2GM* This is the BioCreative II gene mention recognition task aimed at identifying the genes and proteins.
- *BC4CHEMD* This is the BioCreative IV chemical entity mention recognition task aimed at identifying the genes and proteins.
- *BC5CDR* This is the most recent BioCreative V chemical and disease mention recognition task as a combination of BC5CDR-chem and BC5CDR-disease datasets.
- *NCBI-Disease* The NCBI disease dataset was initially introduced for disease name recognition and normalization. It has been widely used for a lot of applications.
- *JNLPBA* This is the 2004 JNLPBA shared task on biomedical entity (gene/protein, DNA, RNA, cell line, cell type) recognition.

### Experiment setup

We denote our model as dictionary-based matching graph network (DBGN). We gatherer biomedical entity dictionaries for three entity types (i.e. genes/proteins, chemicals and diseases) from the Comparative Toxicogenomics Database (CTD)^20^ and the biomedical dataset website (https://github.com/cambridgeltl/MTL-Bioinformatics-2016) . We compare our model with several competitive methods, i.e. MTM^21^, CollaboNet^22^, BERT^23^, BioBERT^11^, and BioBERT with masked manner. Our constructed dictionary consists of 62,351 biomedical domain-specific entities. Note that all methods are already enhanced by conditional random field (CRF)^24^.

### Parameter settings

All the neural network models are trained on one GeForce GTX2080Ti GPU. We use BioBERT pre-trained on PubMed for 1M steps, which is referred as BioBERT v1.1 (+ PubMed). It contains 12 hidden layers and 768 hidden units for each layer. We use Adam^25^ as the optimizer for BioBERT and our model with the learning rate initialized by 0.00001 and 0.001, respectively. Decay rate of the learning is set to 0.98. Except for the influence of decay rate, the learning rate decreases dynamically according to the current step number. Batch shuffling is also applied to the training process.

The hidden size of our basic BiLSTM is 256 and the size of all word embeddings is set to 100. The vocab size of BioBERT is 30,522. The batch size of all model is set to 50. As for regularization, dropout function is applied to word embeddings and the dropout rate is set as 0.1. Besides, we perform L2 constraints over the soft-max parameters and L2-norm regularization is set as 0.0001. We train our model for max to 50 epochs and conduct the same experiment for 10 times with random initialization. We follow the experimental setup in Lee et al. ^11^ and report the average value for all metrics on testing set, where Precision, Recall and Macro-Averaged F1 are adopted as the evaluation metrics. The layer size of BiGCN is set to 2 for all experiments.

### Metrics

We report the performance on testing set. Predicted entities are thought as correct predictions only if they exactly match the golden ones. Based on this principle, we compute Precision, Recall and F1 in a macro-averaged way on all entity types.

16 $$\begin{aligned}{} & {} P=\frac{\sum c_i}{\sum p_i} \end{aligned}$$

17 $$\begin{aligned}{} & {} R=\frac{\sum c_i}{\sum g_i} \end{aligned}$$

18 $$\begin{aligned}{} & {} Macro-F1=\frac{2PR}{P+R} \end{aligned}$$

*i* is the sample index. $p_i$ denotes the number of predicted entities, and $g_i$ denotes the number of golden entities for the *i*-th sample. $c_i$ represents the number of correctly predicted entities.

## Results

### Benchmark performance

In Table 2, the following observations can be obtained: (1). Original BERT does not Lead to a significant improvement in performance. (2). BioBERT improves the performance of all five datasets due to its domain-specific representation ability. (3). The performance improvement of the masked biomedical dictionary approach is minimal because it cannot handle complex situations such as overlapping and nested matching entities. (4). Our model significantly improves the performance and outperforms all other competitive alternatives on BC2GM, BC4CHEMD, BC5CDR, and NCBI-Disease, owing to the application of dictionary-based matching graph. (5). CollaboNet achieves the best performance on JNLPBA because of the employment of external sources. Although BERT and BioBERT cost much time owing to the complex structure, they achieve considerable performance improvements. Our method requires significantly less training time, except for BioBERT which we use as the base encoder.

**Table 2:** Performance and average training time of the baseline neural network models and the proposed model DBGN.

Dataset	Metrics	MTM	CollaboNet	BERT	BioBERT	BioBERT+Masked	DBGN
BC2GM	P	82.1	80.5	81.1	84.3	84.8	**85.7**
	R	79.4	79.0	82.4	85.1	85.4	**90.1**
	F1	80.7	79.7	81.8	84.7	85.1	**87.9**
BC4CHEMD	P	91.3	90.8	91.2	92.8	**93.3**	92.0
	R	87.5	87.0	88.9	91.9	92.1	**96.1**
	F1	89.4	88.9	90.0	92.4	92.7	**94.0**
BC5CDR	P	89.1	*91.2	*87.5	*91.0	91.4	**92.4**
	R	88.5	*90.3	*88.7	*92.9	93.3	**94.5**
	F1	88.8	*90.7	*88.1	*91.9	92.3	**93.4**
NCBI-Disease	P	85.9	85.5	84.1	88.2	88.7	**90.3**
	R	86.4	87.3	87.2	91.3	91.6	**93.2**
	F1	86.1	86.4	85.6	89.7	90.1	**91.7**
JNLPBA	P	70.9	**74.4**	69.6	72.2	72.4	72.9
	R	76.3	83.2	81.2	83.6	83.9	**84.3**
	F1	73.5	**78.6**	74.9	77.5	77.7	78.2
TS	Time(s/b)	1.4	2.0	2.7	2.7	2.8	3.1

Scores in the asterisked (*) cells are obtained in the experiments that we conducted, and these scores are not reported in the original papers. The best scores from these experiments are in bold, TS means training speed.

### Layer size study

**Figure 4:**
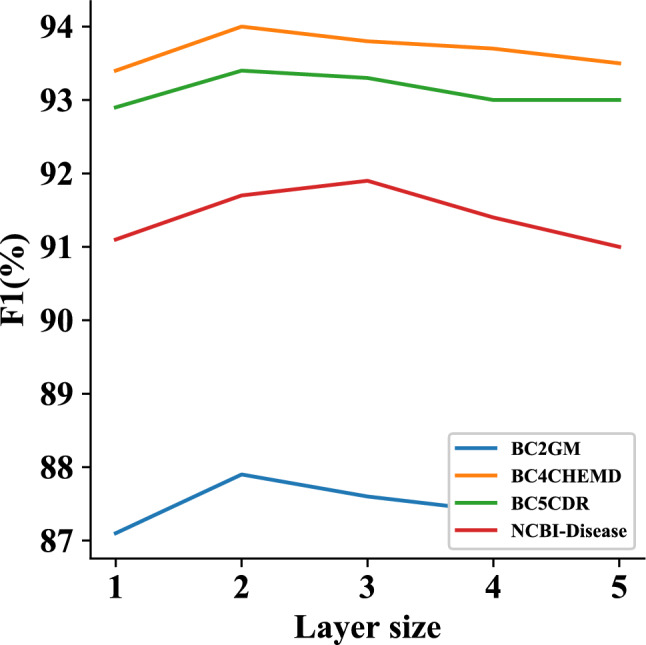
A performance curve by the layer size of BiGCN on four datasets.

Figure 4 shows that the model achieves the best performance with a layer size of two for BC2GM, BC4CHEMD, and BC5CDR, and three for NCBI-Disease. We exclude JNLPBA from this analysis as its performance variance is not obvious. If the layer size is too low, the information may not be fully propagated. Conversely, if the layer size is too large, the model may overfit. Therefore, the layer size should be determined based on the specific experimental results.

### Ablation study

There are four major ablation conditions used in Table 3: -BiGCN, -Residual Connection (RC), -Fuse Layer (FL) and -BiLSTM. -BiGCN means that we remove the backward graph and use only a single GCN. -RC means that we remove the residual connection for every GCN layer. -FL means that we remove the fuse layer for two GCNs and only combine them in the last GCN layer. -BiLSTM means that we remove the BiLSTM layer and only use BioBERT to encode input tokens. As shown in Table 3, we can conclude that BiGCN accounts for the most significant performance improvement, owing to its ability to capture both forward and backward information. FL also contributes to the performance, demonstrating that fusing two GCNs in every GCN layer is better than using them separately. RC, on the other hand, does not noticeably improve the results, but it can significantly reduce the training epoch number required to reach convergence, BiLSTM improves predictive performance through its ability to better capture bidirectional long-range dependencies in sequences.

**Table 3:** The statics of four ablation results on five datasets. RC means Residual Connection and FL means Fuse Layer.

Dataset	DBGN	–BiGCN (only GCN)	–RC	–FL	–BiLSTM
BC2GM	87.9	85.8	87.6	86.7	87.3
BC4CHEMD	94.0	92.8	93.8	93.2	93.5
BC5CDR	93.4	92.4	93.3	92.8	92.5
NCBI-Disease	91.7	90.2	91.5	90.8	91.2
JNLPBA	78.2	77.7	78.2	77.9	78.0

### Case study

Table 4 reports three typical cases. In case 1, masked manner and our model output right label sequences owing to the fact that ‘T-PLL’ is in the dictionary. In case 2, masked manner obtains an overlong and wrong entity owing to an incorrect mask sequence. In case 3, only our model produces the right output. BioBERT generates a relatively short entity due to the lack of the dictionary information, while masked manner produces an overlong entity due to the misleading of the longest masked sequence.These results demonstrate that our method not only leverages dictionary information but also intelligently selects appropriate sub-matching entities to avoid mistakes caused by complex matching situations.

**Table 4:** The results of three typical cases.

	Models	Examples
Case 1	golden	Two of seventeen mutated T - PLL samples had a previously reported A - T allele .
	BioBERT	Two of seventeen mutated T - PLL samples had a previously reported A - T allele .
	Masked manner	Two of seventeen mutated T - PLL samples had a previously reported A - T allele .
	DMGN	Two of seventeen mutated T - PLL samples had a previously reported A - T allele .
Case 2	golden	The ability of VHL - negative RCC cancer cells to exit the cell cycle and enter G0 / quiescence in low serum
	BioBERT	The ability of VHL - negative RCC cancer cells to exit the cell cycle and enter G0 / quiescence in low serum
	Masked manner	The ability of VHL - negative RCC cancer cells to exit the cell cycle and enter G0/quiescence in low serum
	DMGN	The ability of VHL - negative RCC cancer cells to exit the cell cycle and enter G0 / quiescence in low serum
Case 3	golden	Mutated in Angelman syndrome patients who lack 15q11 - q13 deletions or chromosome 15 paternal uniparental disomy .
	BioBERT	Mutated in Angelman syndrome patients who lack 15q11 - q13 deletions or chromosome 15 paternal uniparental disomy .
	Masked manner	Mutated in Angelman syndrome patients who lack 15q11 - q13 deletions or chromosome 15 paternal uniparental disomy .
	DMGN	Mutated in Angelman syndrome patients who lack 15q11 - q13 deletions or chromosome 15 paternal uniparental disomy .

## Conclusions

We propose a dictionary-based matching graph network for biomedical named entity recognition. The proposed approach utilizes the dictionary-based matching graph instead of a simple masked manner, and outperformed state-of-the-art systems and several strong neural network models on benchmark BioNER datasets. We also demonstrate detailed analysis that the strong performance is achieved by the BiGCN module with only a slight increase in training time, and demonstrate that the large performance gains of our approach mainly come from the matching graph.

Finally, we highlight several possible directions to improve our model in future works. First, this method is actually suitable for many similar NLP applications, such as relation extraction and question answering. We can improve the performance of other tasks by applying this method accordingly. Second, by further resolving the entity boundary and type conflict problems, we could build a coherent system for recognizing multiple types of biomedical entities with high performance and efficiency.

## Data Availability

Our dataset access is open. Details of the dataset can be found online at https://github.com/cambridgeltl/MTL-Bioinformatics-2016/tree/master/data.
